# Effect of In Vitro Digestion on Water-in-Oil-in-Water Emulsions Containing Anthocyanins from Grape Skin Powder

**DOI:** 10.3390/molecules23112808

**Published:** 2018-10-29

**Authors:** Weili Xu, Yang Yang, Sophia Jun Xue, John Shi, Loong-Tak Lim, Charles Forney, Guihua Xu, Bio Sigui Bruno Bamba

**Affiliations:** 1 Department of Food Science and Engineering, School of Chemistry and Chemical Engineering, Harbin Institute of Technology, Harbin 150001, China; 15776399795@163.com; 2 Guelph Research and Development Centre, Agriculture and Agri-Food Canada, 93 Stone Road West, Guelph, ON N1G 5C9, Canada; jun.xue@agr.gc.ca (S.J.X.); guihuaxu@uoguelph.ca (G.X.); bamba_bio@upgc.edu.ci (B.S.B.B.); 3 Department of Food Science, University of Guelph, Guelph, ON N1G 2W1, Canada; llim@uoguelph.ca; 4 Kentville Research and Development Centre, Agriculture and Agri-Food Canada, Kentville, NS B4N 1J5, Canada; charles.forney@agr.gc.ca

**Keywords:** anthocyanins, W/O/W double emulsions, controlled release, in vitro digestion, soybean protein isolate

## Abstract

The effects of in vitro batch digestion on water-in-oil-in-water (W/O/W) double emulsions encapsulated with anthocyanins (ACNs) from grape skin were investigated. The double emulsions exhibited the monomodal distribution (d = 686 ± 25 nm) showing relatively high encapsulation efficiency (87.74 ± 3.12%). After in vitro mouth digestion, the droplet size (d = 771 ± 26 nm) was significantly increased (*p* < 0.05). The double W_1_/O/W_2_ emulsions became a single W_1_/O emulsion due to proteolysis, which were coalesced together to form big particles with significant increases (*p* < 0.01) of average droplet sizes (d > 5 µm) after gastric digestion. During intestinal digestion, W_1_/O droplets were broken to give empty oil droplets and released ACNs in inner water phase, and the average droplet sizes (d < 260 nm) decreased significantly (*p* < 0.05). Our results indicated that ACNs were effectively protected by W/O/W double emulsions against in vitro mouth digestion and gastric, and were delivered in the simulated small intestine phase.

## 1. Introduction

Anthocyanins (ACNs) extracted from grape/berry are a group of water-soluble pigments belonging to the flavonoids classes [1]. The predominant ACNs in Nature are glycosides of polyhydroxy- and polymethoxy-derivatives of flavylium (2-phenylbenzopyryliurn) salts [2]. Epidemiologic studies suggested that ACNs have several health-promoting functions in the prevention of many chronic diseases, such as cardiovascular disease, diabetes, arthritis, and cancer [3,4]. According to the latest research, the ACN extracts from plant materials such as berry pomace have stronger antioxidant properties and are more effective in altering the development of obesity since they contain diverse phenolic compounds that have synergistic effects, compared to pure phenolic compounds [5,6,7]. The bioactivity of ACN extracts makes them one of the most effective ingredients used in functional foods. However, extracted ACNs are easily degraded during processing and storage in the presence of light, high temperature, alkaline conditions, oxygen or enzymes [8]. Moreover, their bioactivity could be lost when processing through the human digestive system [9,10].

Microencapsulation has long been used to protect sensitive and labile ingredients against degradation and interaction with other ingredients, and mask their odour and bitter taste, and it has become increasingly important in the food industry [11,12,13]. Furthermore, microencapsulation processes focusing on berry pomace extract as carriers, instead of free single pure compounds, can effectively enhance the bioactivity, functionality, absorption rate, and bioavailability [13]. A growing trend is the delivery of a mixture of bioactive ingredients through microencapsulation as it is recognized that a wide range of therapeutic effects are obtained from the synergistic effects among different bioactives. Water-in-oil-in-water (W_1_/O/W_2_) double emulsions, which can be generated by dispersing a water-in-oil (W_1_/O) single emulsion in a continuous aqueous phase (W_2_), have been proved to be one of the best microencapsulation techniques for the protection and controlled release of hydrophilic bioactive compounds [13,14,15,16,17]. Meanwhile, the in vitro digestion model has gained much attention as a tool for understanding the physicochemical changes and controlled release of encapsulated components [18,19]. Previous studies have demonstrated that the physicochemical properties of the inner aqueous phase, oil phase, and emulsifiers all play vital roles in the encapsulation efficiency, stability, and release of encapsulated bioactive components through the gastrointestinal (GI) passage [15,20,21].

Although several studies have been conducted on the digestion behaviors of double-layer microcapsules [14,15,17,22,23,24,25,26,27,28,29], the change on the microstructure of W/O/W double emulsions was still not fully characterized. There is limited information about sequential digestion of W/O/W emulsions from the mouth through to the GI tract, especially comparative studies with or without lipase in the small intestine digestion model. Soybean protein isolate (SPI) has been used as a emulsifying agent and wall material for the production of oil-in-water emulsions [30]. However, the use of SPI as an emulsifier in ACNs–rich W/O/W double emulsions as well as its digestion behavior were not reported. Therefore, the main objectives of this study were to prepare water-extracted ACNs-loaded W_1_/O/W_2_ double emulsions with SPI solution as the outer water phase (W_2_) to achieve controlled release and increase the stability of ACNs in the GI tract. The morphology, storage stability, microstructure, particle size, ζ-potential, controlled delivery, and antioxidant activity of ACNs–rich emulsions after sequential digestion with α-amylase, pepsin and pancreatin (or lipase) using batch model digestion were characterized.

## 2. Results and Discussion

### 2.1. Morphology and Storage Stability of the W_1_/O/W_2_ Emulsion

**Figure 1:**
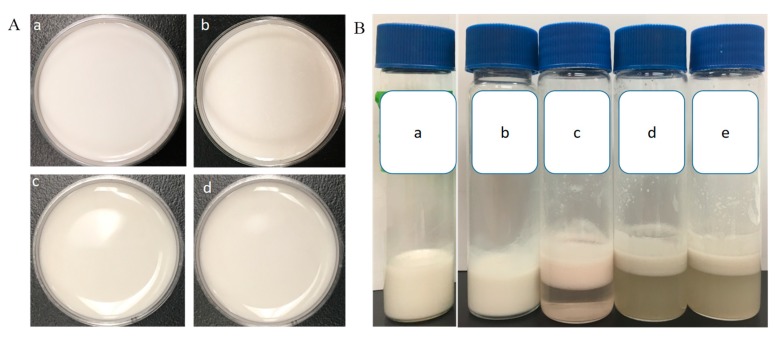
Photographs of the W/O/W emulsions. (**A**) Storage stability of the double emulsion at 4 °C. (**a**) fresh preparation; (**b**) 3-week; (**c**) 4-week; (**d**) 8-week. (**B**). the creaming stability of the emulsion after in vitro digestion and 24-h storage at 25 °C. (**a**) control group; (**b**) after mouth digestion; (**c**) after gastric digestion; (**d**) after intestinal digestion with pancreatin; (**e**) after intestinal digestion (with pancreatin + lipase).

**Figure 2:**
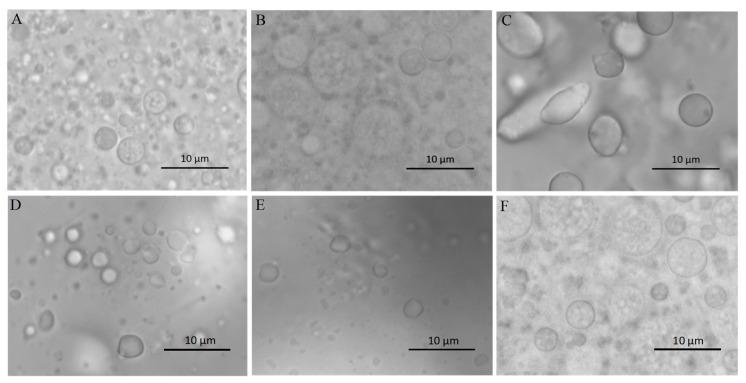
Photomicrographs of W/O/W emulsions from in vitro digestion model and after storage. (**A**) before digestion; (**B**) mouth digestion for 10 min; (**C**) gastric digestion for 0.5 h; (**D**) intestinal digestion (with pancreatin) for 2 h; (**E**) intestinal digestion (with pancreatin + lipase) for 2 h; (**F**) W/O/W emulsions after 8 weeks at 4 °C.

The morphology and stability of W/O/W double emulsions-encapsulated ACNs extracts were evaluated. As shown in Figure 1Aa–d, the W/O/W emulsions were homogeneous, and the surfaces were smooth. Microscopic analysis revealed that numerous small droplets were in the internal phase of the large globules (Figure 2A), indicating a Type-B W/O/W multiple emulsions according to the Florence and Whitehill classification [31]. The emulsion samples stored at 4 °C for 3, 4, and 8 weeks were homogenous, without visible signs of flocculation, coalescence, or creaming (Figure 1Ab–d). The droplets were spherical in shape and evenly distributed in the system (Figure 2F). After storage at 4 °C for 8 weeks, the absolute value of the ζ-potential (−38.23 ± 0.31 mV) was decreased obviously compared with the fresh emulsion (−42.03 ± 0.26 mV), which suggested that the stability of W/O/W double emulsions decreased with the storage time. The ζ-potential value represents the electrostatic repulsive interaction between particles in order to prevent aggregation [16,32]. After in vitro GI digestion with enzyme and storage at 4 °C for 24 h, obvious phase separation in the W_1_/O/W_2_ emulsions was observed (Figure 1Bc–e), indicating that the emulsions became unstable due to the aggregation of lipid droplets.

### 2.2. Effects of In Vitro Simulated Digestion on W_1_/O/W_2_ Emulsion Microstructure

**Figure 3:**
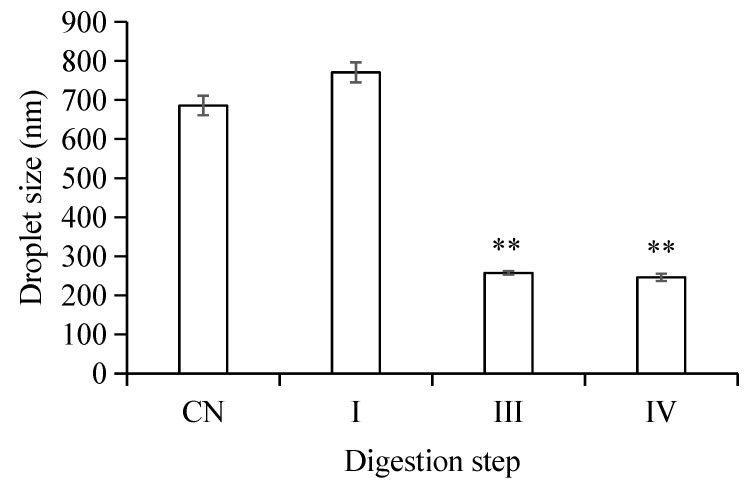
Mean particle diameters of W/O/W double emulsions droplets in different in vitro digestion phases. (CN) control, before digestion; (I) mouth digestion for 10 min; (III) intestinal digestion (with pancreatin) for 2 h; (IV) intestinal digestion (with pancreatin + lipase) for 2 h. Values are mean ±SD, n = 3. ** *p* < 0.01 versus the control group.

**Figure 4:**
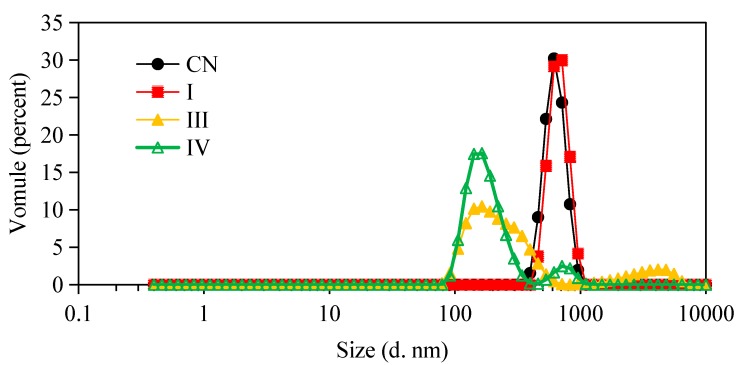
Particle size distributions of W/O/W double emulsions containing ACNs before and after in vitro digestion. (CN) control, before digestion; (I) mouth digestion for 10 min; (III) intestinal digestion (with pancreatin) for 2 h; (IV) intestinal digestion (with pancreatin + lipase) for 2 h.

The microstructure changes of the W_1_/O/W_2_ emulsions at different stages of in vitro digestion were evaluated using microscopy (Figure 2) and dynamic light scattering (Figure 3 and Figure 4). The average droplet diameter of original W/O/W emulsions was 686 ± 25 nm (PDI = 0.449 ± 0.202) (Figure 3), and their droplet size distribution was in the range of 390 to 960 nm (Figure 4). After mouth digestion (Stage I), the droplet size (771 ± 26 nm) was significantly increased (*p* < 0.05) with a monomodal size distribution (Figure 3 and Figure 4), and the micrographs indicated that α-amylase affected the emulsion microstructure (Figure 2B). The average droplet size of the emulsions was more than 5 µm after gastric digestion (Stage II) (Figure 2C, Figure 3 and Figure 4), which could be attributed to the digestion of the proteins at the interfaces of double-emulsions by pepsin. The double emulsion droplets collapsed, leading to the release of W_1_/O droplets, which were coalesced to give larger droplets during gastric digestion (Figure 2C). This could be the main reason for the phase separation shown in Figure 1Bc. Changes in emulsion structure were not observed in the control group without pepsin (data not shown). In contrast, previous studies [14,28,29] showed the double emulsions were stable during artificial gastric incubation, although Xiao et al. [17] found that the majority of double emulsion droplets prepared with soybean oil containing PGPR as oil phase (O), and Kafirin protein as external water phase (W2) collapsed and released the inner water phase (ACN) after gastric digestion. The inconsistency between our results and those of Frank et al. [14], Giroux et al. [28], Shima et al. [29], and Xiao et al. [17] may be associated with the different chemical composition and pH of the simulated gastric juice, as well as the outer water phase. When the emulsions were processed from the simulated gastric to the intestinal conditions, the average droplet size (d = 257 ± 5 at Stage III; and d = 246 ± 9 nm at Stage IV) decreased significantly (*p* < 0.01) (Figure 3 and Figure 4). The micrographs confirmed that W_1_/O droplets were broken due to the hydrolysis of oil phase, and inner-water phase was released when pancreatin or pancreatin + lipase was introduced (Stage III or Stage IV) (Figure 2D,E), which was consistent with the results reported by Frank et al. [14] and Shima et al. [29].

### 2.3. Effects of In Vitro Simulated Digestion on ζ-Potential of W_1_/O/W_2_ Emulsions

**Figure 5:**
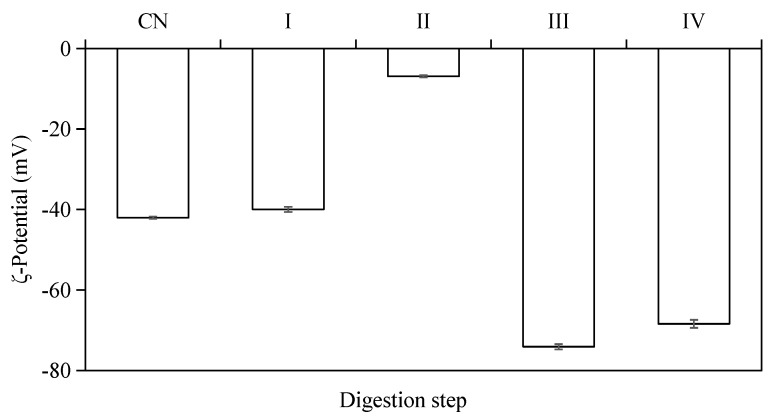
ζ-potentials of W/O/W double emulsions containing ACNs after the in vitro digestion process. (CN) control, before digestion; (I) mouth digestion for 10 min; (II) gastric digestion for 0.5 h; (III) intestinal digestion (with pancreatin) for 2 h; (IV) intestinal digestion (with pancreatin + lipase) for 2 h.

The electrical charge (i.e., ζ-potential) on the emulsion droplets was monitored during the different stages of in vitro digestion (Figure 5). The results indicated that all W/O/W double-emulsion samples had negative ζ-potential values at different stages of the in vitro digestion. The original emulsions (pH = 6.6–6.8) exhibited strong negative charge (−42.03 ± 0.26 mV), which was mainly depended by the outer emulsifiers and pH of the emulsion. The ζ-potential of emulsion droplets decreased to −39.97 ± 0.62 mV after simulated mouth digestion (pH = 6.6–6.8), and the emulsions had the lowest ζ-potential value (−6.88 ± 0.27 mV) after the gastric digestion (pH = 2.0). The surface layers of these droplets were damaged by digestive enzymes and some negative charge from the residues in interface protein was lost. 

A low negative ζ-potential was obtained at pH 2 for the emulsion, which indicated the droplets were easily aggregated together after gastric digestion. When the emulsions were processed through the simulated small intestine conditions (pH = 6.8), the absolute value of the ζ-potential (−74.10 ± 0.65 mV at stage III, −68.4 ± 1.00 mV at stage IV) became even higher than the original emulsions. The changes in the electrical charges of emulsion droplets can be attributed to: (1) changes in pH and ionic strength of solution; (2) adsorption of charged particles from the digestive juices onto the emulsifier coating [33,34,35].

### 2.4. Effects of In Vitro Simulated Digestion on Rheologicalogy of W_1_/O/W_2_ Emulsions

**Figure 6:**
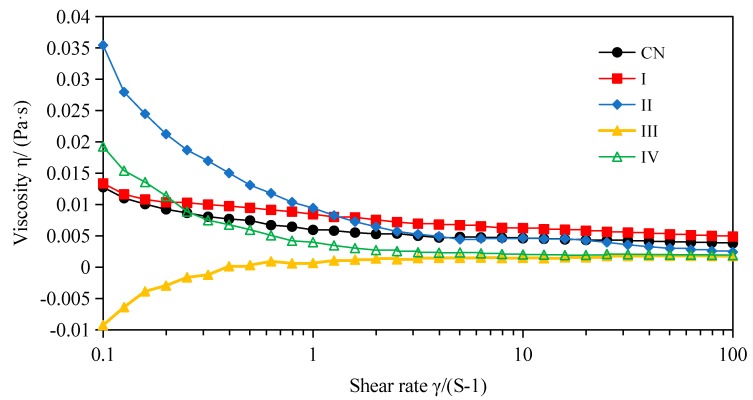
Viscosity-rate of shear curves for W_1_/O/W_2_ emulsions prior to and following in vitro mouth, gastric and intestinal digestion at room temperature (25 °C). (CN) control, before digestion; (I) mouth digestion for 10 min; (II) gastric digestion for 0.5 h; (III) intestinal digestion (with pancreatin) for 2 h; (IV) intestinal digestion (with pancreatin + lipase) for 2 h.

The emulsion viscosity depends on the nature of the droplet interactions and the degree of aggregation at a given mass concentration of emulsions [36]. The viscosity of the emulsions in different stages of in vitro digestion was tracked using a rheometer (Figure 6). The viscosity of the W_1_/O/W_2_ double emulsion stabilized by SPI was decreased with increasing shear rate, which indicated the emulsion had non-Newtonian fluid behavior at shear rates of 0.1–100 1/s. The results were consistent with the findings of Mahmood et al. [37] and Wang et al. [38]. However, it has been reported that double emulsions displayed Newtonian fluid behavior [39]. These different results may be due to the type and concentration of the emulsifiers used, as well as the volume of the dispersive phase. The viscosity of double emulsion after mouth digestion decreased slowly, similar to the initial emulsion, which indicated that α-amylase had no significant effect on the structure of the double emulsion. Obvious shear thinning behavior was found in the W_1_/O/W_2_ emulsions after in vitro gastric and intestinal digestion with pancreatin. In contrast, a shear thickening phenomenon could be observed after simulated intestinal digestion with pancreatin + lipase. Both phenomena might be due to the deformation and/or break-up of the emulsion droplets after in vitro digestion [40].

### 2.5. Release of ACNs from the W_1_/O/W_2_ *Emulsions* during In Vitro Digestion

**Figure 7:**
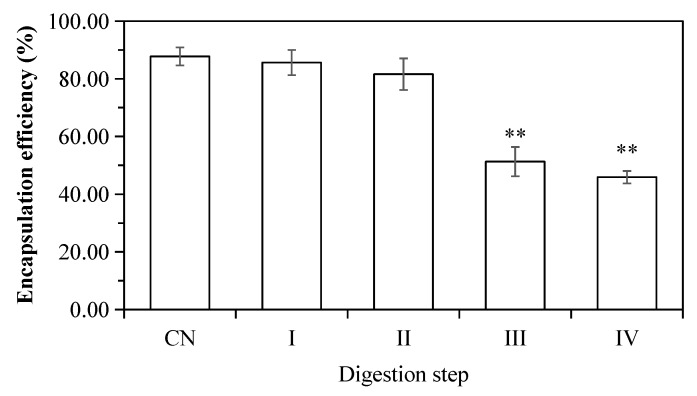
The encapsulation efficiency of ACNs into W_1_/O/W_2_ double emulsions. (CN) control, before digestion; (I) mouth digestion for 10 min; (II) gastric digestion for 0.5 h; (III) intestinal digestion (with pancreatin) for 2 h; (IV) intestinal digestion (with pancreatin + lipase) for 2 h. Values are mean ± SD, n = 3. ** *p* < 0.01 versus the control group.

The release of encapsulated ACNs during in vitro digestion is presented in Figure 7. The ACN encapsulation efficiency in initial W_1_/O/W_2_ emulsions is 87.74 ± 3.12% (*w*/*w*). ACNs were not released and remained in the inner phase of double emulsions after mouth digestion (Stage I), which also confirmed that α-amylase did not destroy the microstructure of the emulsion droplets. The CAN release rate (~6%, *w*/*w*) was increased after gastric digestion (Stage II). The release of the inner W_1_ phase in the W_1_/O droplets to the exterior water phase during enzymatic digestion might be caused by the diffusion or coalescence of the W_1_ droplets with the surrounding water phase [14,41,42,43]. Similar findings were also reported by Frank et al. [14], Oidtmann et al. [44], and Flores et al. [45]. However, Xiao et al. [17], and Andrade et al. [22] reported that the inner aqueous phase (W_1_) of W/O/W double emulsions was mostly released at the gastric stage due to the collapse of droplet structure. Shima et al. [29] indicated that the double emulsions were not affected by the artificial enzyme-free stomach fluid and the encapsulation efficiency was unchanged. The previous studies confirmed that the addition of pepsin destroyed emulsion structure leading to subsequent releases of ACNs.

In the present study, ACNs were mostly released during simulated intestinal digestion, at which the ACNs release rate was 36.47 ± 3.25% under intestinal digestion with pancreatin (Stage III), and 41.86 ± 3.75% at Stage IV (with pancreatin + lipase). The main reason could be that the oil layer of W_1_/O droplets was hydrolyzed and ACNs were released after pancreatin or with pancreatin + lipase digestion. Our results were in agreement with those described by Frank et al. [14], Flores et al. [46], Giroux et al. [28] and Shima et al. [29]. In contrast, Aditya et al. [25] reported that more than 45% of curcumin was released from the W/O/W double emulsions within 1 h after in vitro enzyme-free intestinal digestion. The discrepancy might be associated with chemical composition of the artificial digestive fluid, encapsulation method, types of wall material, or physicochemical properties of the inner aqueous phase [22]. It is worth noting that the release rate of ACNs determined by HPLC may be lower than the actual value. The reasons could be attributed to (1) the interactions between ACNs and peptides [47]; (2) released ACNs were unstable and degraded faster under small intestinal conditions [48].

### 2.6. Antioxidant Activity Changes during In Vitro Digestion

**Figure 8:**
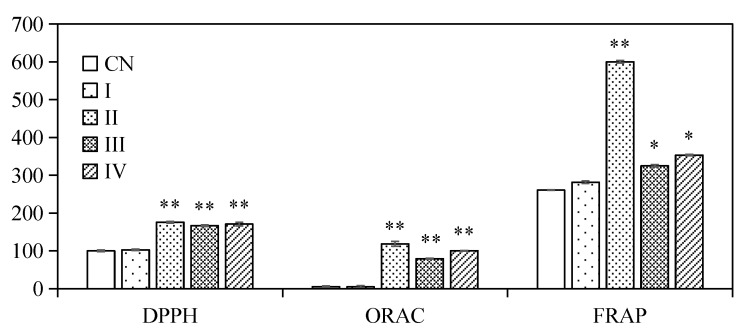
Antioxidant activity of W/O/W micro-emulsions containing ACNs by DPPH, ORAC and FRAP assays before and after in vitro digestion. (CN) control, before digestion; (I) mouth digestion for 10 min; (II) gastric digestion for 0.5 h; (III) intestinal digestion (with pancreatin) for 2 h; (IV) intestinal digestion (with pancreatin + lipase) for 2 h. DPPH assay, values are expressed as %; ORAC assay, values are expressed as μmol/L Trolox equivalent; FRAP assay, values expressed as μmol/L ascorbic acid equivalent. Values are mean ± SD, n = 3. * *p* < 0.05 versus the control group, ** *p* < 0.01 versus the control group.

The antioxidant activity of encapsulated ACNs after in vitro digestion was evaluated, and presented in Figure 8. The results showed that there was no obvious change on the DPPH, ORAC and FRAP values of the emulsions during mouth digestion compared with the initial emulsion. DPPH, ORAC, and FRAP values increased significantly (*p* < 0.05) after gastric and intestinal digestion steps. There were still 81.59 ± 5.50% of ACNs trapped in the oil droplets, however, the highest antioxidant activities were identified during simulated gastric digestion with pepsin (Stage II)*.* The partially reason could be attribute to the hydrophilic nature of ACNs, which enabled the ACN molecules to diffuse readily into hydrophilic media [25]. Moreover, it might also be partially contributed by the antioxidant peptides, which have been confirmed from SPI hydrolyzed by pepsin [49]. Our results were consistent with previous reports of Flores et al. [45], Flores et al. [46], Betz et al. [50] and Oidtmann et al. [44], which showed microcapsules were able to stabilize ACNs and maintain high antioxidant activity throughout simulated GI digestion. In contrast, Cofrades et al. [23] reported that in vitro gastric and intestinal digestion significantly reduced the antioxidant activity of the double emulsions and gelled double emulsions due to the loss of bioactive compounds.

The highest release amount and stronger antioxidant activity of encapsulated ACNs into W/O/W double emulsion were detected during intestinal digestion. Furthermore, there is no obvious difference in the antioxidant capacity of the samples digested by intestinal juice with pancreatin (Stage III) or pancreatin + lipase (Stage IV). Our results confirmed that the pancreatin and lipase played a key role in the targeted delivery of loaded components in double emulsion stabilize by SPI to the small intestine.

## 3. Materials and Methods

### 3.1. Materials and Chemicals

Grape skin powder was provided by Joseph’s Natural Products Inc. (Niagara-on-the-Lake, ON, Canada). SPI (non-denatured) (87.11%) was purchased from Nutrabio Co. (Middlesex, NJ, USA). α-Amylase from porcine pancreas, pepsin from porcine gastric mucosa, pancreatin and lipase from porcine pancreas, and porcine bile salt were purchased from Sigma-Aldrich (St. Louis, MO, USA). Corn oil was purchased from a local market. ACN standards (i.e., delphinine chloride, cyanidin chloride, pelargonidin chloride and malvidin chloride) were purchased from Indofine Chemical Company Inc. (Somerville, NJ, USA). Hydrochloric acid, citric acid, and sodium benzoate were purchased from Sigma Scientific (Oakville, ON, Canada). Polyglycerol polyricinoleate (PGPR, 4175) was kindly donated by Palsgaard Inc. (Morris Plains, NJ, USA). 6-Hydroxy-2,5,7,8-tetramethyl-chroman-2-carboxylic acid (Trolox), 2, 2, azobis-(2-methylpropionamidine) dihydrochloride (AAPH), 2,2-diphenyl-1-picrylhydrazyl (DPPH), L-ascorbic acid, fluorescein, and phenylmethanesulfonyl fluoride (PMSF) were purchased from Sigma-Aldrich (St. Louis, MO, USA). Sodium chloride and HPLC-grade solvents (including methanol, isopropanol, formic acid, hexane, and ethanol) were purchased from Caledon Laboratories (Georgetown, ON, Canada).

### 3.2. Preparation of SPI Solutions and Grape Skin Extracts

The SPI powder was dissolved in deionized water (2.5%, *w*/*w*), stirred for 2 h and stored at 4 °C for 12 h to allow complete hydration. Grape skin powder (20 g) was extracted twice in 600 mL of 50% (*v*/*v*) methanol at 50 °C for 1 h under ultrasonication, and then filtered through Whatman Grade No. 1 filter paper. Filtrates were combined, concentrated by a rotary evaporation at 40 °C. The final extracts (without methanol) were stored at −20 °C for further uses.

### 3.3. Preparation of the W/O/W Double Emulsions

The double emulsions were referred as W_1_/O/W_2_, where W_1_ is the inner water phase, O is the oil phase, and W_2_ is the outer water phase. The primary W_1_/O emulsion was created by dropwise addition of 30 g grape skin extract into 70 g oil system (including 66 g corn oil and 4 g PGPR), and homogenization at 10,000 rpm for 10 min using a Polytron PT 2500 E (Kinematica AG, Luzern, Switzerland). The W_1_/O emulsion (20 g) was added to the outer-phase solution (80 g), and the mixture was homogenized at 6000 rpm for 5 min to obtain a coarse W/O/W emulsion. The solution was then passed through a microfluidizer (ATS Scientific Inc., Burlington, ON, Canada) at 500 Bar to obtain the W_1_/O/W_2_ double emulsions.

### 3.4. Simulated In Vitro Digestions

The in vitro batch digestion followed a previously reported method with minor modification [21]. Briefly, four separated batch of tests were conducted with a control (CN, before digestion). Stage I, to study the effect of mouth digestion: double emulsion (6 mL) was mixed with 120 μL α-amylase at a concentration of 60 U/mL; the mixtures were incubated for 10 min at 37 °C in a water bath shaker at 100 rpm/min, and PMSF with the final concentration of 174 mg/mL was added to stop the digestion. Stage II, to study the effect of gastric digestion: after the same α-amylase incubation procedures as Stage I, 3 mL phosphate buffer saline (PBS) was added, and the pH was adjusted to 2 with 1 M HCl (0.35 mL); porcine pepsin (94.7 µL) with a final concentration of 1.3 mg/mL was mixed using the vortex and incubated for 30 min under the same conditions, and PMSF was added to stop the digestion. Stage III, to study the effect of intestinal digestion with pancreatin: after the same procedures as Stage II, the pH of the mixture was adjusted to 5.8 by dropwise addition of 1 M NaOH (0.38 mL); pancreatin (100 µL) with a final concentration of 0.175 mg/mL, porcine bile salt (100 µL) with a final concentration of 1.1 mg/mL, and 100 µL deionized water was added, respectively; the pH was adjusted to 6.8 by adding 1 M NaOH, and the digestate was incubated in a shaking water bath at 37 °C for another 2 h; PMSF with a final concentration of 0.174 mg/mL was added to stop the digestion process. Stage IV, to study the effect of intestinal digestion with lipase (100 µL): the same procedures were conducted as Stage III, but lipase with a final concentration of 0.03 mg/mL was mixed with pancreatin and porcine bile salt during hydrolysis. After digestions, 2 mL Milli-Q water was added to 2 mL digest, and mixed on a vortex. The diluted samples were filtered using a 30,000-MWCO Amicon-Ultra 15-Centrifugal Filter device (Millipore, Eschborn, Germany) at a centrifuge speed of 6,000 rpm for 30 min (5810R, Brinkman Instruments Inc., Westbury, NY, USA). The filtrate was decanted and collected for further analysis. A blank (without adding sample) was used as a reference, and incubated under the same conditions. All experiments were carried out in triplicate.

### 3.5. Microscopy Observation

Photographs were taken to evaluate the changes in surface morphology of the emulsions. The microstructure of the emulsions was measured using an optical microscope (Axio Imager A2, Zeiss, Jena, Germany) at a 100× magnification connected to a AxioCam MRC5 camera (Zeiss).

### 3.6. Creaming Stability

The emulsions collected in different digestion steps were transferred into test tubes, tightly sealed with a plastic cap, and stored at 25 °C for 24 h. The photos of the emulsions were taken by a digital camera.

### 3.7. Particle Size and ζ-Potential

The emulsions were collected in the test tubes after each digestion step, and diluted with Milli-Q water to 0.5% (*w*/*w*). The measurements of Zeta-potential and particle size of the diluted emulsions were carried out with a Zetasizer (Nano-ZS90, Malvern Instruments, Worcestershire, UK) at 25 °C. Each ζ-potential measurement was calculated from the average of ten readings from each sample. Optical microscopy was also applied to particle size measurement.

### 3.8. Determination of ACNs by HPLC

The grape skin extract (60 μL), equivalent to the amount in 1 mL W/O/W emulsions, was diluted to 5 mL with 3.3 mL of 5 M HCl and 1.7 mL of Milli-Q water. The solution was held at 100 °C for 60 min to hydrolyze ACNs to anthocyanidins. After cooling down, the solution was filtered through 0.45 μm syringe filters. HPLC analysis was performed using Agilent 1100 systems equipped with a photodiode-array detector (PAD) (Agilent Technologies, Waldbornn, Germany) and a C_18_ (150 mm × 4.6 mm i.d. × 5 μL) analysis column (Dikma Inc., Beijing, China). The temperature was maintained at 25 °C. Gradient elution was performed using 10% (*v*/*v*) formic acid (solution A) and 100% methanol (solution B) as follows: 0–20 min, linear gradient from 95% A/5% B to 40% A/60% B; 20–23 min, isocratic elution at 40% A/60% B; 23–24 min, linear gradient from 40% A/60% B to 95% A/5% B; 24–28 min, isocratic elution at 95% A/5% B. Flow rate was 0.8 mL/min, and the injection volume was 100 μL. External standards (i.e., delphinine chloride, cyanidin chloride, pelargonidin chloride, and malvidin chloride) quantified at 520 nm, and calibration curves were established based on the peak areas (y = 10.977x − 42.692, R^2^ = 0.9935; accuracy: 0.01 μg/mL).

### 3.9. Release of ACNs from the W/O/W Emulsion

The release rates of ACNs from the W/O/W double emulsions were determined by HPLC. Briefly, 2 mL W/O/W emulsions were diluted with 2 mL Milli-Q water and centrifuged using a 30,000-MWCO Amicon-Ultra 15-Centrifugal Filter device at 6000 rpm for 30 min. The filtrate were collected for HPLC analysis to evaluate ACNs encapsulation efficiency. Encapsulation efficiency (EE %) of ACNs was calculated using the following equation:

EE% = (G0 − G1)/G0 × 100(1)

where G1 is the free non-entrapped amount of ACNs in the emulsion, and G0 is the total quantity of ACNs added initially during preparation. The release of ACNs (R %) was calculated as fellow:

R% = 100 − EE%(2)

### 3.10. Antioxidant Activities Evaluation

The antiradical activity (DPPH assay) of the samples was determined spectrophotometrically in a UV-Vis plate reader by monitoring the disappearance of DPPH at 517 nm, following the procedure described by Li and coworkers [51]. The radical scavenging activity was expressed as Trolox equivalents. The ORAC assay was conducted based on a previously reported method [21]. The results were expressed as μmol Trolox equivalents by using the standard curve calculated for each assay. The FRAP assay was conducted according to a previously reported procedure [21,22]. Absorbance readings were taken using a visible-UV microplate reader (Power Wave XS2, Bio-Tek Instruments Inc., Winooski, VT, USA) at 593 nm. The antioxidant activities were expressed as μmol ascorbic acid equivalents.

### 3.11. Statistical Analysis

Results were expressed as means ± SD of three independent extractions. One-way analysis of variance (ANOVA) was used to compare the results. All statistical analyses were performed using SPSS 14.0 (SPSS Inc., Chicago, IL, USA).

## 4. Conclusions

In this study, in vitro digestion behavior of ACN-loaded W_1_/O/W_2_ double emulsions stabilized by PGPR and SPI was investigated. The average droplet diameters of original W/O/W emulsions were 686 ± 25 nm. After gastric digestion, the average droplet size increased significantly (*p* < 0.01) with lowest ζ-potential value (−6.88 ± 0.27 mV) compared with other in vitro digestion stages, while the droplet size decreased significantly (*p* < 0.01) during simulated intestine digestion with the highest ζ-potential value (−74.10 ± 0.65 mV at Stage III, −68.40 ± 1.00 mV at Stage IV). The double-layer structure of the emulsions has been destroyed leading to the release of W_1_/O droplets, which were coalesced to give largest droplets during gastric digestion, and the encapsulated ACNs were targetedly delivered to the simulated small intestinal step. W/O/W double emulsions effectively protected ACNs against simulated mouth and gastric digestion, which have proven to be promising microcarrier systems to control the release of bioactive compounds in food and pharmaceutical products.
